# Variation in actuarial senescence does not reflect life span variation across mammals

**DOI:** 10.1371/journal.pbio.3000432

**Published:** 2019-09-13

**Authors:** Guillaume Péron, Jean-François Lemaître, Victor Ronget, Morgane Tidière, Jean-Michel Gaillard

**Affiliations:** Université de Lyon, Université Lyon 1, CNRS, Laboratoire de Biométrie et Biologie Évolutive UMR5558, Villeurbanne, France; University of Newcastle upon Tyne, UNITED KINGDOM

## Abstract

The concept of actuarial senescence (defined here as the increase in mortality hazards with age) is often confounded with life span duration, which obscures the relative role of age-dependent and age-independent processes in shaping the variation in life span. We use the opportunity afforded by the Species360 database, a collection of individual life span records in captivity, to analyze age-specific mortality patterns in relation to variation in life span. We report evidence of actuarial senescence across 96 mammal species. We identify the life stage (juvenile, prime-age, or senescent) that contributes the most to the observed variation in life span across species. Actuarial senescence only accounted for 35%–50% of the variance in life span across species, depending on the body mass category. We computed the sensitivity and elasticity of life span to five parameters that represent the three stages of the age-specific mortality curve—namely, the duration of the juvenile stage, the mean juvenile mortality, the prime-age (i.e., minimum) adult mortality, the age at the onset of actuarial senescence, and the rate of actuarial senescence. Next, we computed the between-species variance in these five parameters. Combining the two steps, we computed the relative contribution of each of the five parameters to the variance in life span across species. Variation in life span was increasingly driven by the intensity of actuarial senescence and decreasingly driven by prime-age adult mortality from small to large species because of changes in the elasticity of life span to these parameters, even if all the adult survival parameters consistently exhibited a canalization pattern of weaker variability among long-lived species than among short-lived ones. Our work unambiguously demonstrates that life span cannot be used to measure the strength of actuarial senescence, because a substantial and variable proportion of life span variation across mammals is not related to actuarial senescence metrics.

## Abstract 2

Looking at human life history, differences in aging rate seem to be the main source of variation in longevity. But analysis of accurate individual lifespan records across 96 nonhuman mammalian species in captivity suggests that early-life processes and factors that are independent of age play a prominent role, apparently greater than aging.

## Introduction

The extreme range of variation in animal life span has puzzled biologists for a very long time [1–5]. Analyzing life span is challenging because it is an integrative trait that results from the cumulative effect of changes in instantaneous survival probability [6], and factors of variation in life span are often age specific [7–10]. Life span is yet frequently used to measure the intensity of actuarial senescence (usually defined as the observed increase in mortality hazards with age) [11–14]. This practice implies that variation in life span is mostly caused by variation in the intensity of actuarial senescence across species. However, with the recent increase in long-term individual-based studies [15], we now have empirical evidence that life span is not a good proxy for actuarial senescence across individuals in a range of species [16–21]. From a theoretical viewpoint as well, actuarial senescence should have a limited demographic impact compared with mortality earlier in life [22,23], which expectedly decouples the variation in the rate of actuarial senescence from the variation in life span. Thus, some life span variation across species should be caused by either age-independent mortality (i.e., factors that affect all age classes in the same way) or early-life mortality (e.g., factors that mostly affect young adults) [24]. To the best of our knowledge, these hypotheses have never been tested empirically, and the relative contributions of the successive life stages to variation in life span across species have never been quantified.

**Fig 1:**
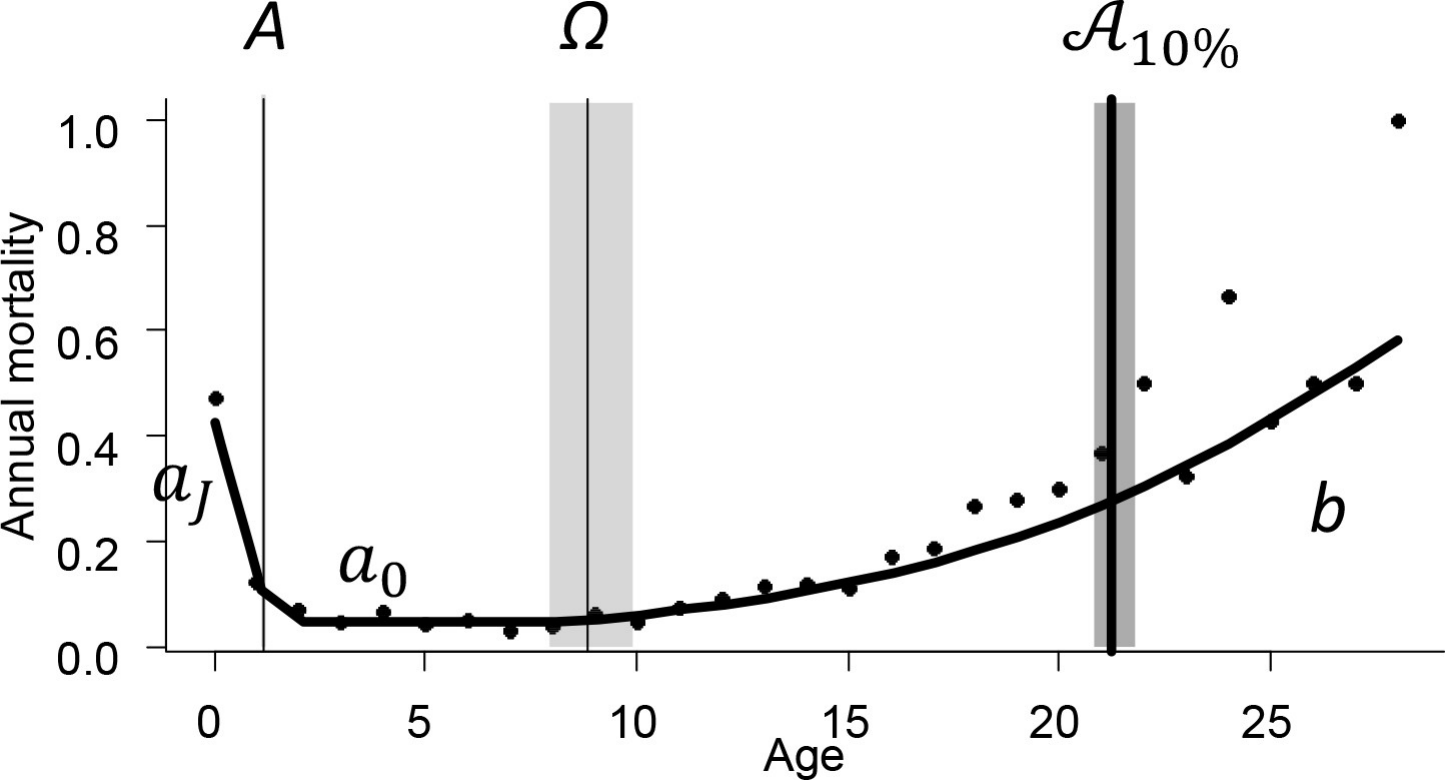
The five-parameter mortality model that we used in this study, featuring *A*: the duration of the juvenile stage, Ω: the age at the onset of actuarial senescence, *a*_*J*_: the mortality hazard during the juvenile stage, *a*_0_: the mortality hazard during the prime-age adult stage, and *b*: the rate of actuarial senescence after the onset. In addition, we represent the 10% life span $A_{10\%}$: the predicted age at which 90% of the cohort is dead. The black dots represent the empirical mortality rates: proportion among individuals that reached age *x* of those that died before age *x* + 1. The data for this example come from captive lionesses (*Panthera leo*). The data for this figure are accessible online as S1 Data.

We took advantage of a unique dataset of accurate individual longevities from mammals that were born and died in zoological institutions to fill that gap. We performed the first comparative analysis of the relative contributions of successive life stages as quantified by age-specific mortality curves (Fig 1). We looked into how these contributions varied across species. Importantly, we included all causes of mortality, including those that are traditionally categorized as “intrinsic” and “extrinsic” [25]. In theory, there is a potential confounding effect of “extrinsic” causes of mortality on the estimation of actuarial senescence because variation in “extrinsic” mortality can influence the number of individuals that live to experience senescence and because mortality from different causes may exhibit different age-specific patterns [26]. However, in most cases, including the present study, the different factors of mortality operate in a compensatory way, meaning that supressing one factor of mortality—for example, predation—may not eventually improve the life expectancy [27], in part because exposure to extrinsic factors of mortality over evolutionary times has shaped the intrinsic deterioration in performance with age [11] and in part because extrinsic causes of mortality are rarely fully additive. For example, predators often select the weaker individuals with intrinsically lower chances of survival. For this reason, it was not realistic for us to try and focus only on confirmed cases of organismal failure, and we analyzed all the deaths irrespective of mortality cause (but see [28]). We acknowledge that our study animals did not live in a controlled or common garden experiment, that not all individuals died of old age, and that extrinsic factors such as weather or rearing conditions likely varied across species and institutions. We thus strictly estimated the age at which mortality rates start to increase and the rate of that increase and interpreted these parameters as corresponding to actuarial senescence. Because age-specific patterns of mortality vary along the slow–fast continuum of life histories going from large-sized species with late maturation and low fecundities to small-sized species with early maturation and high fecundities [29], we expected the following:

1. Late-acting deleterious mutations are subjected to weaker selective pressure when adult mortality is high, and therefore, variation in the intensity of actuarial senescence should be less constrained in short- than long-lived species [23,30,31]. Actuarial senescence patterns should thus be more variable in short- than long-lived species (i.e., in small than in large species because we used body mass to rank species on the slow–fast continuum, cf. Material and methods section). However, the resulting contribution of actuarial senescence to variation in life span also depends on the sensitivity of life span to actuarial senescence, which we will quantify along the slow–fast life history continuum.
2. Adult mortality during the prime-age stage (i.e., when mortality is minimum), rather than juvenile mortality or the duration of the juvenile stage, should account for the remaining variance not explained by actuarial senescence. This is because a small change in annual prime-age mortality has a larger cumulative impact in long- than in short-lived species [32] and also because the population growth rate (i.e., the population-average fitness sensu Fisher [33]) of long-lived species is much less sensitive to a given variation in juvenile mortality than to the same variation in prime-age mortality [34]. We thus expected the contribution of prime-age mortality to variation in life span to increase from short- to long-lived species (i.e., from small to large species because we used body mass to rank species on the slow–fast continuum, cf. Material and methods section).

## Results

### Age-specific mortality curves and allometric relationships

**Fig 2:**
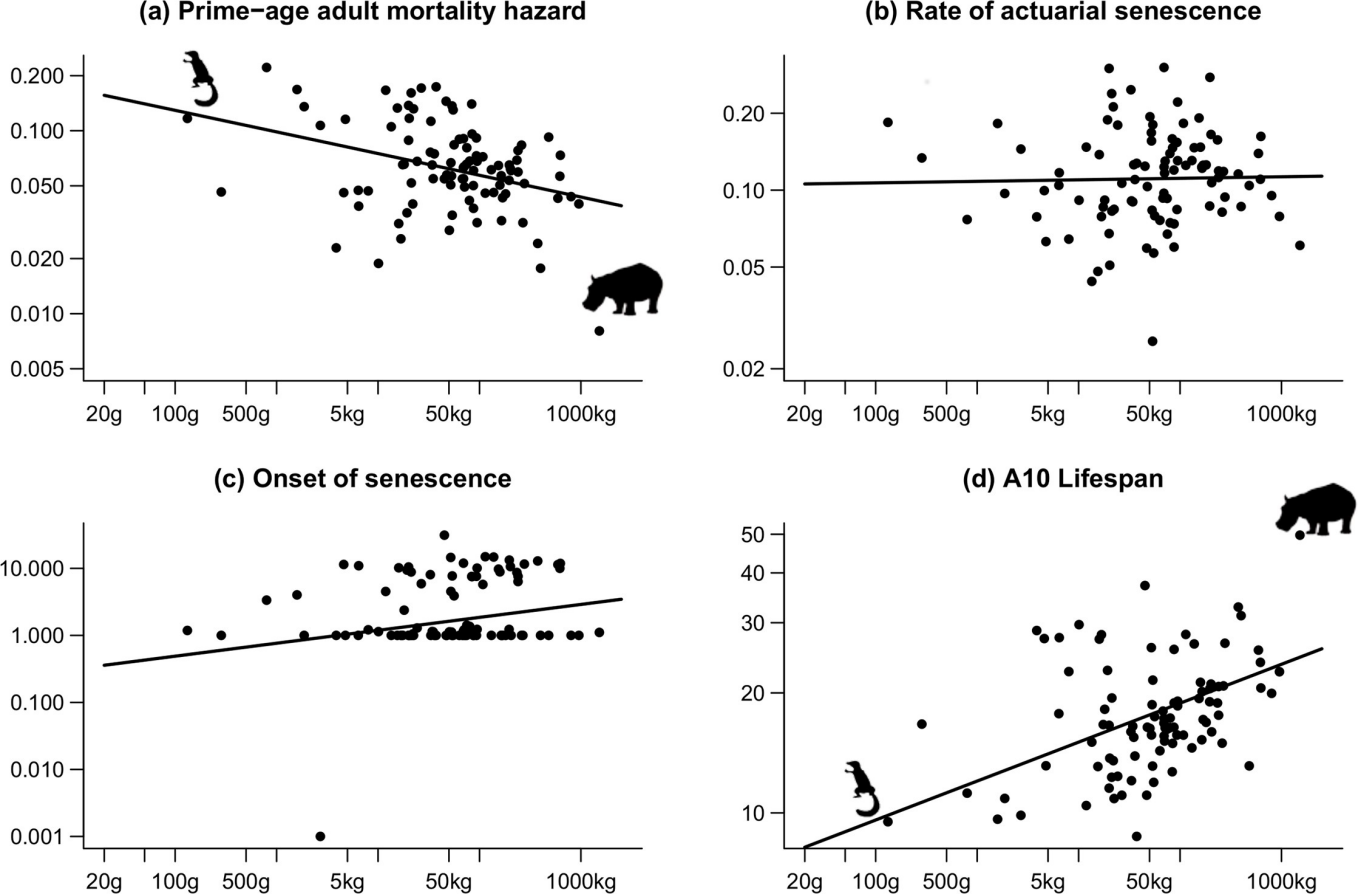
Species-specific estimates of the (a) prime-age mortality hazard, (b) the rate of actuarial senescence, (c) the onset of actuarial senescence (in years), and (d) the 10% life span (in years), plotted against the species-specific body mass. The regression lines are from phylogenetic generalized least square regressions. Note the log scale on both axes. The pictograms represent the lightest and heaviest species in the sample (tree shrew *Tupaia glis* and hippopotamus *Hippopotamus amphibius*). The data for this figure are accessible online as S2 Data.

The 10% life span correlated to body mass (slope of 0.10 ± 0.02 on the log-log scale; Fig 2D), supporting the general expectation that large species live on a slower lane than small species and that body mass reliably reflects the species-specific rank along the slow–fast continuum of life histories. More precisely, prime-age adult mortality decreased from small to large species (slope of −0.12 ± 0.03 on the log-log scale; Fig 2A), whereas senescence started later (slope of 0.19 ± 0.08 on the log-log scale; Fig 2C) but was not detectably slower once it started (slope of 0.006 ± 0.03 on the log-log scale; Fig 2B). The frequency of detection of a prime-age stage increased from small to large species (slope of 0.06 ± 0.03 on the logit-log scale). Large species were less likely than small species to enter the senescent stage immediately upon entering adulthood. Phylogenetic inertia was detected for the 10% life span and the onset of actuarial senescence ($\hat{\lambda }$ = 0.99 and 0.59, respectively; *P* < 0.001), which are both biological times, but not for the other parameters, which are rates.

### Elasticity of life span

**Fig 3:**
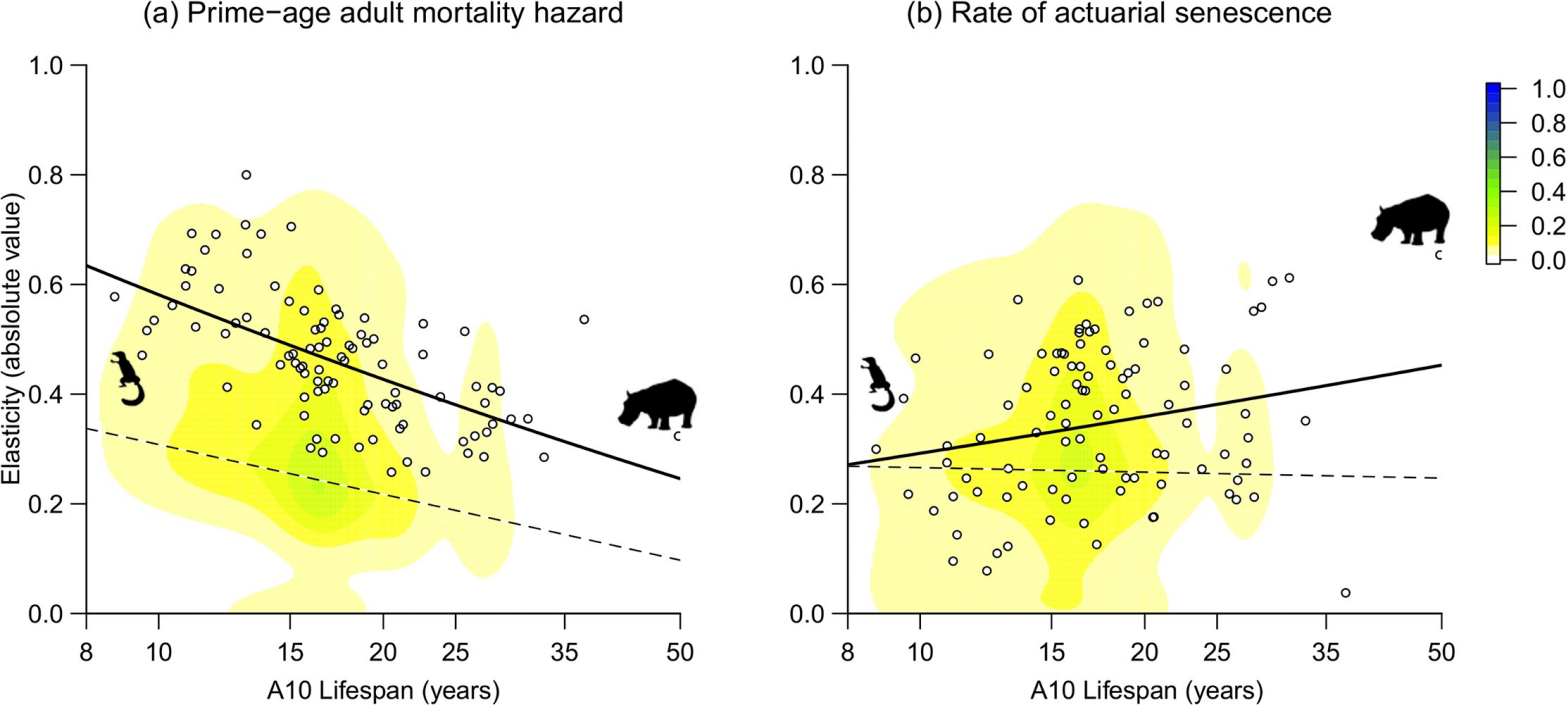
Elasticity of the life span to (a) the baseline mortality hazard and to (b) the rate of actuarial senescence. The color scale represents the distribution of elasticity by chance (see “Conformity test” in the Material and methods section). The solid lines represent the interspecific regression across observed values. The dashed lines indicate the life span–specific mode of the expected distribution by chance. The pictograms represent the lightest and heaviest species in the sample (tree shrew *T*. *glis* and hippopotamus *H*. *amphibius*). The shortest-lived, leftmost point on this graph is *Saiga tatarica*. The data for this figure are accessible online as S3 Data.

In the majority of species, the observed elasticities of life span to the prime-age adult mortality and to the rate of actuarial senescence were above the modal expected values of elasticity under the null hypothesis of the conformity test (Fig 3). The elasticities of life span to the other three parameters were much lower (all <0.1). This means that the shape of the observed age-specific mortality curves consistently conferred more influence on life span to prime-age adult mortality and to the rate of actuarial senescence than to the other three parameters and than expected by chance.

The distance between the observed and modal expected elasticity of life span to prime-age mortality decreased from short-lived to long-lived species (slope of −0.09 ± 0.03 on the log-log scale; Fig 3A). This means that the effect of changing the prime-age mortality was closer to the expectation by chance in long-lived than in short-lived species. The distance between the observed and modal expected elasticity of life span to the rate of actuarial senescence increased from short-lived to long-lived species (slope of 0.11 ± 0.04 on the log-log scale; Fig 3B). This means that the effect of changing the rate of actuarial senescence increased more than expected by chance when the pace of life slowed down.

### Between-species variation in the shape of the mortality curve

The between-species variation in the five parameters of the mortality curve consistently decreased with increasing body mass (heteroscedasticity test: all *P* < 0.01). Between the subset of light species and the subset of heavy species, the coefficient of variation decreased by a factor 1.5 for prime-age mortality, 1.3 for the rate of actuarial senescence, and 1.6 for the duration of the prime-age period.

### Decomposition of the variance in life span

**Fig 4:**
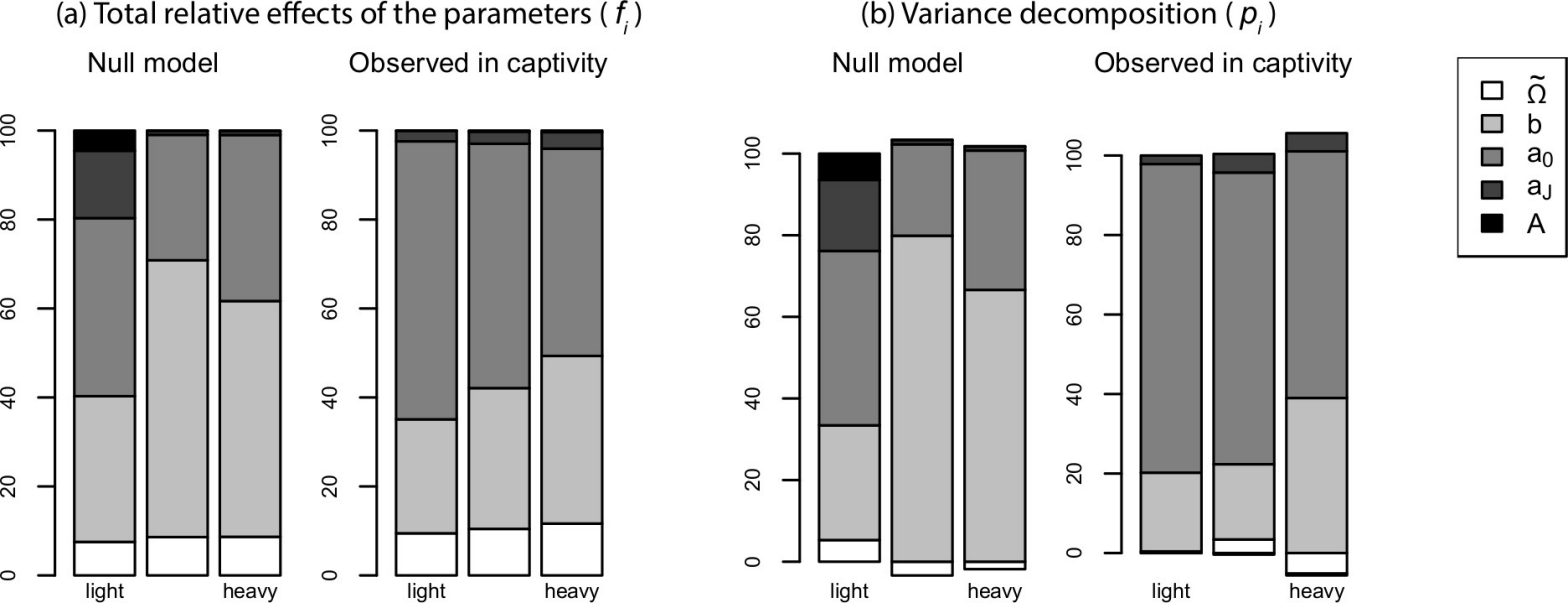
Decomposition of the variance in female life span across 96 mammalian species divided in three terciles of body mass (cf. Material and method section). (a) Effect sizes obtained by summing both positive and negative (co)variance terms. (b) Variance decomposition sensu stricto, allowing some parameters to offset the contribution of others via the covariance terms. All the observed contributions of mortality parameters to variation in life span were statistically different from the expectation under the null model described in the "Conformity test" section of the main text. *A* is the duration of the juvenile stage, *a*_*J*_ is the juvenile mortality hazard, *a*_0_ is the baseline prime-age adult mortality hazard, *b* is the rate of actuarial senescence, and $\tilde{\Omega }$ is the duration of the prime-age stage. The data for this figure are accessible online as S4 Data.

Overall, the summed contributions of actuarial senescence parameters (i.e., rate and onset) varied between 35% in the tercile of lighter species and 49% in the tercile of heavier species (Fig 4). In other words, actuarial senescence was not the main contributor to observed variation in life span across mammal species, whereas the null model predicted it would be (Fig 4).

The contribution of the rate of actuarial senescence to variation in life span was smaller than expected by chance (*f*_*i*_ = 26%, 32%, and 38% in each tercile versus expected 33%, 62%, and 53%; *P* < 0.001; Fig 4). By contrast, the contribution of the prime-age mortality to the variation in life span was larger than expected by chance (*f*_*i*_ = 62%, 55%, and 47% versus expected 40%, 28%, and 37%; *P* < 0.001; Fig 4). The contribution of the duration of the prime-age period was moderate and similar in the two species subsets (*f*_*i*_ = 9%, 10%, and 11% versus expected 7%, 9%, and 9%; Fig 4). Juvenile parameters had almost no contribution to variation in life span, which was expected for the heavier species but not for the lighter species (Fig 4).

Actuarial senescence nevertheless played a larger role in the life span variation of heavy than in light species (Fig 4). These differences between heavy and light species were mostly driven by the way the elasticities varied with life span (Fig 3). Indeed, the elasticities of senescence and prime-age parameters varied in ways that fully explained their relative contributions to life span variation (Fig 3 and Eq 3), whereas, by contrast, the parameter values became less variable as body mass increased, meaning that the parameter variability (“cv” in Eq 3 in the Material and method section) could not explain the differences between the contributions of senescence and prime-age parameters.

Lastly, although we did not make any prediction regarding the role of covariance terms, it is noteworthy that the only large negative *p*_*i*_ variance component was the contribution of the duration of the prime-age period in the heavier species (Fig 4). This was because of a positive correlation term between the duration of the prime-age period and the mortality hazard during the prime-age stage combined with a relatively low variance in the duration of the prime-age period and high variance in prime-age adult mortality (Fig 2). This means that the contribution of the duration of the prime-age period to variation in life span was mostly driven by the variation in the prime-age mortality hazard via the covariance term, rather than directly via the variance term. This is one of the reasons why, in our view, the *f* values yielded a clearer picture of the relative role of the parameters than the *p* values (Fig 4).

## Discussion

### Life span does not reliably measure actuarial senescence

Variability in the way mortality increased with age never accounted for more than half of the variance in life span across 96 mammal species, which sampled a large array of life history strategies from short-lived tree shrews to long-lived hippopotamuses. Thus, we demonstrate that using life span metrics, such as mean or maximum longevity, to quantify actuarial senescence is not reliable and should be discouraged. For example, when MacRae and colleagues [35] reported a link across species between DNA repair transcriptomes and maximum life span or Gomes and colleagues [36] found a link between telomere length and maximum life span, they did not compulsorily demonstrate any effect on mortality acceleration with age or actuarial senescence per se. The expression of DNA repair genes and the effect of telomere length could instead operate on prime-age, baseline mortality and yield the same observed correlations with life span [24].

All animals in our study were born and died in zoological institutions, which in effect put them in an intermediate environment between strictly controlled lab conditions (in which most deaths could be attributed to “intrinsic” factors) and wild conditions (in which most deaths are caused by an interplay between “extrinsic” and “intrinsic” mortality factors that would have shaped the life history of the study species). This represents an obvious limit to the extent to which our inference can be generalized into evolutionary theory or can be used to discuss the proximal, physiological mechanisms of aging. However, in captivity, baseline mortality rates should be buffered against environmental variation [37,38] because many factors of mortality are controlled for (e.g., predation, food scarcity, most pathogens). Decreasing the variance in baseline mortality should have reduced the contribution of baseline mortality to variation in life span. By contrast, we found that baseline mortality is a major contributor to variation in life span, which makes our findings particularly robust.

### A narrow path toward long life span?

In the life history theory, the elasticity of fitness to variation in a trait quantifies the strength of selection on that trait, as shaped by the life history strategy [23,34,39]. For example, individual fitness in long-lived species is more responsive to changes in adult survival than to changes in fecundity. This is interpreted as evidence for stronger selection against factors limiting adult survival than against factors limiting fecundity [34,40]. In the disposable soma theory, this further translates into an increase in resource allocation to repair mechanisms at the expense of a decrease in allocation to reproductive effort [41]. More proximally, two theories, the reliability theory of aging [42] and the bet-hedging theory of life history [43], both predict selection for decreased failure risk, at the level of individual cells, organs, or somatic functions in the reliability theory and at the integrative and behavioral levels (risk avoidance) in the bet-hedging theory. We focused here on life span, which is one component of fitness, and analyzed the elasticity of life span to five parameters that describe the age-specific mortality curve. In keeping with life history theory, the prime-age mortality hazard exhibited the largest elasticities and therefore constituted the pathway through which selection on life span would operate most effectively. The second most effective pathway was the rate of actuarial senescence, and the importance of this pathway increased in long-lived species, whereas the importance of prime-age mortality, counterintuitively, decreased (Fig 3A versus 3B). We furthermore found significant departures from the expectation of elasticity by chance. We interpret these patterns as the signature of life history evolution. Indeed, many scenarios that we considered possible in the simulations—for example, low prime-age mortality associated to early and fast senescence—are biologically impossible when senescence is molded by natural selection [23]. Short-lived species exhibited later onsets of senescence than expected from their longevity, probably as a by-product of their iteroparous life history strategy [40,44]. This explained that the elasticity of their life span to the rate of actuarial senescence was lower than that of long-lived species (Fig 3B). Long-lived species exhibited low elasticity to prime-age mortality (Fig 3A) because the latter was already minimized to a large extent. They exhibited higher elasticity to the rate of actuarial senescence than expected (Fig 3B) because they cannot delay the onset of senescence to the point at which the rate of senescence loses its influence, which was a possibility in the simulations.

More generally, the way the components of Eq 3 (in the Material and method section) varied along the slow–fast continuum of life histories further informs the evolution of life histories. First, the parameters of the age-specific mortality curve became less variable as body mass increased and the pace of life slowed down. This result aligns neatly with the observation that, in long-lived species, within a given population, variation in adult mortality is drastically reduced compared with juvenile mortality [45]. Although the relative roles of canalization sensu stricto as opposed to direct directional selection are not definitively quantified [46], the amount of within-species variation in stage-specific mortality and its potential influence on fitness are clearly negatively correlated, both theoretically and empirically [47–50]. Here, the same pattern shows up at the between-species level, too: the parameters that contributed the most to the variation in life span were also the least variable across species and the closest to the expectation by chance. Age-specific mortality curves appeared to be less diverse in large than in small species. Nevertheless, actuarial senescence played a larger role shaping the life span of long- than of short-lived species. This was apparent both at the elasticity level (Fig 3B) and in the variance decomposition (Fig 4), indicating that the increase in the elasticity of life span to the rate of actuarial senescence compensated the canalization of the rate of actuarial senescence. These observations lay the foundation for a general demographic framework that explains a range of emerging patterns in life history variation across species and environments. Thus, long-lived species already avoid mortality risks to the point that their prime-age mortality rates are already as close to zero as possible in the wild, meaning that the change from natural to captive conditions can hardly be beneficial [51]. By contrast, short-lived species trade mortality risks for reproductive investment so that when these mortality factors are artificially controlled [37,38] or when allocation to reproduction is artificially prevented [52], the life span increases. Lastly, the onset of senescence might be more variable than the rate of senescence [9], in line with the differences in elasticity that we report, but we acknowledge this inference is sensitive to the realism of the piecewise model of mortality that we proposed. Overall, our findings support the existence of a narrow pathway toward long life spans for mammalian species, which leads to a reduced diversity of mortality patterns among long-lived species and more influence for actuarial senescence than expected.

## Material and methods

### Datasets

We restricted the analyses to females. Demographic data were obtained from the Zoological Information Management Systems (ZIMS) database from the Species360 organization (https://www.species360.org/) under the 2017–06 data agreement. This unique organization collates and standardizes information from approximately 6.5 million individuals belonging to >21,000 species (mostly vertebrates) across >1,000 zoological and aquaria institutions in over 90 countries. For each species, following previous works [38,53], we considered that captive individuals constitute a metapopulation representing how the species performs in captivity. We did not access information about the cause of death and hereafter study life span variation irrespective of the cause of death (as commonly done in life span studies [54]). The database documents the precise age at death, at least for individuals born in captivity, which we exclusively focused on. Because of the participatory nature of the data gathering by the ZIMS, some records are incomplete—i.e., it is not always possible to decipher whether an individual’s death failed to be reported or it is still alive. To circumvent that issue, we only analyzed the data from extinct cohorts, which are cohorts for which all individuals have been documented as dead or are past the maximum age ever reported for the species. From an initial set of 144 species [38,55], we selected those for which at least 25 females were documented to have reached the age of 1 year, at least five had reached the predicted age at which 90% of an average cohort would be dead (denoted ${\hat{A}}_{10\%}$; Fig 1; explained below), and the model-selection procedure (explained below) yielded evidence of actuarial senescence. The final dataset featured 96 species (S5 Data). This relatively stringent selection procedure ensured that the observed role of senescence in shaping life span was not influenced by the inclusion of species that do not exhibit any actuarial senescence and that the sample size to estimate the life span and actuarial senescence was large. On average, 220 study individuals per species reached the age of 1 year (range: 35–1,247 for *Alcelaphus buselaphus* and *P*. *leo*, respectively) and 18 individuals reached the age of ${\hat{A}}_{10\%}$ (range: 5–98, also for *A*. *buselaphus* and *P*. *leo*).

### A slightly modified Siler model of age-specific mortality

The Siler model [56] describes the “bathtub” mortality curves that are widespread among animals (e.g., Fig 1 in [42], p. 16). More specifically, the Siler model features three life stages: juvenile, prime-age, and senescent. It successfully captures the key aspects of mammalian mortality curves—namely, mortality is highest during the juvenile stage [57], decelerates to reach a plateau of minimal mortality during the prime-age stage, and eventually accelerates with age during the senescence stage in an exponential way (see [31] for a review). To yield this bathtub shape, the Siler model uses a product of three survival functions, each of them being defined and operating throughout the entire lifetime [56]. In other words, the three risks are competing at each age in the Siler model. Alternatively, we can view the mammalian mortality curve as the succession of three separate stages. We opted for the latter formulation. We used a piecewise formulation of the bathtub model (Fig 1 and Eq 1, where *l*(*x*) is the proportion of individuals in a cohort that are still alive at age *x* in years). In addition to clearly separating the contributions of the three life stages, this approach allowed us to more straightforwardly simplify the survival model in a data-driven way by comparing the fit of models without one or two of the life stages to the full three-stage model. Moreover, we took advantage of the fact that our inference did not depend on the shape of mortality within the juvenile stage to characterize the juvenile stage using two parameters only: the duration of the juvenile stage and the average mortality hazard over the juvenile period. Compared with the original Siler model, we spared one parameter: the rate at which mortality decreases with increasing age over the juvenile period. This simplification was actually critical because the data were provided with a resolution of 1 year, meaning that the juvenile period was often documented by only one or two data points. Our formulation had five parameters as follows:

$$\{\begin{array}{ll} x\in \left[0;A\right] & l\left(x\right)=e^{-a_{J}x}\\ x\in ]A;\Omega ] & l\left(x\right)=e^{-a_{J}A}e^{-a_{0}\left(x-A\right)}\\ x\in ]\Omega ;+\infty [ & l\left(x\right)=e^{-a_{J}A}e^{-a_{0}\left(\Omega -A\right)}e^{a_{0}/b}exp\left[-a_{0}/b\cdot e^{b\left(x-\Omega \right)}\right] \end{array}$$ (1)

with *a*_*J*_ being the average mortality hazard during the juvenile stage, *A* the duration of the juvenile stage, *a*_0_ the prime-age (minimal, baseline) mortality hazard that applies between age *A* and Ω, Ω the age at the onset of actuarial senescence, and *b* the (exponential) rate of actuarial senescence. Note that any change in *a*_0_ applies to all ages beyond the juvenile period. We also hereafter use $\tilde{\Omega }=\Omega -A.\tilde{\Omega }$ measures the duration of the prime-age stage. In summary, *a*_*J*_ and *A* represent the juvenile stage, *a*_0_ represents the prime-age stage, and *b* and Ω represent the senescent stage.

### Parameter estimation

We estimated the five parameters of the mortality curve separately for each species using maximum likelihood estimation. We first tried to simplify the model of Eq 1 to find the smallest number of parameters that still satisfactorily described the observed mortality curve. For this purpose, we implemented a model-selection procedure comparing the fit of the full model (M1 as shown in Eq 1) to the fit of seven simpler nested models (S6 Data): (1) we removed the prime-age stage to obtain M2 (Ω = *A*, which corresponds to the Gompertz model with onset at age *A*); (2) we removed the juvenile stage to obtain M3 (*A* = 0); (3) we fixed the duration of the juvenile stage to 1 year to obtain M4 (*A* = 1); (4) we removed both the juvenile and prime-age stages to obtain M5 (Ω = *A* = 1); (5) we removed the senescent stage to obtain M6, which corresponds to a two age-class model (i.e., juveniles versus adults); and (6) we kept mortality constant with age to obtain M7. For each species, we selected the model with the lowest Akaike information criterion corrected for sample size (AICc). When M6 or M7 was selected with a difference of more than two AICc points, we concluded a lack of support for actuarial senescence.

### Measuring life span

The maximum recorded life span in a longevity dataset typically depends on the sample size, meaning that the maximum recorded life span is a potentially biased metric of life span [58,59]. Instead, we used the age at which 90% of a cohort was predicted to be dead, also called the “10% life span”: $A_{10\%}=\left\{x|l\left(x\right)=0.1\right\}$ [58,59]. From Eq 1, it follows that

$${\hat{A}}_{10\%}=\{\begin{array}{ll} if\;l\left(\hat{A}\right)\leq 0.1 & {\hat{A}}_{10\%}=-\frac{\text{log}\left(0.1\right)}{{\hat{a}}_{J}}\\ if\;l\left(\hat{A}\right)>0.1\;\text{and}\;l\left(\hat{\Omega }\right)\leq 0.1 & {\hat{A}}_{10\%}=\hat{A}-\frac{\log\left(0.1\right)+{\hat{a}}_{J}\hat{A}}{{\hat{a}}_{0}}\\ if\;l\left(\hat{\Omega }\right)>0.1 & {\hat{A}}_{10\%}=\hat{\Omega }+\frac{1}{\hat{b}}\text{log}\left[1-\frac{\hat{b}}{{\hat{a}}_{0}}\left(\log\left(0.1\right)+{\hat{a}}_{J}\hat{A}+{\hat{a}}_{0}\hat{\Omega }\right)\right] \end{array}$$ (2)

### Decomposition of the variance in life span

Hereafter, by convenience, we replace the notation $\left\{a_{J},\;a_{0},b,A,\;\tilde{\Omega }\right\}$ with {*θ*_1_,*θ*_2_,*θ*_3_,*θ*_4_,*θ*_5_,}. For each parameter *θ*_*i*_, we computed $s_{i}\left(\overset{-}{\theta_{i}}\right)$, the sensitivity of life span to that parameter, by deriving Eq 2 against *θ*_*i*_ and evaluating that partial derivative at the mean value of *θ*_*i*_ [60]. $s_{i}\left(\overset{-}{\theta_{i}}\right)$ measures the rate of change in life span with (infinitesimal) changes in parameter *θ*_*i*_. We also computed $e_{i}\left(\overset{-}{\theta_{i}}\right)$, the elasticity of life span to parameter *i*, by deriving the log of Eq 2 against the log of $\theta_{i}.e_{i}\left(\overset{-}{\theta_{i}}\right)$measures the change in life span caused by an infinitesimal proportional change in parameter *θ*_*i*_. An elasticity of 0.5 means that a 1% change in the focal parameter yields a 0.5% change in life span, all else being equal [60]. Next, we computed all the *var*(*θ*_*i*_), the variances across species in parameter *θ*_*i*_, cv(*θ*_*i*_), the coefficients of variation, and *ρ*_*ij*_, the coefficients of correlation across species between *θ*_*i*_ and *θ*_*j*_.

Following the first-order Taylor decomposition of the life span taken at its mean value across species, the variance in life span caused by (co)variance between any two parameters *θ*_*i*_ and *θ*_*j*_ was then *C*_*ij*_ from Eq 3. We rescaled these contributions to obtain the net relative contribution of parameter *i*, *p*_*i*_, and the total effect size of parameter *i*, *f*_*i*_ (both defined in Eq 3). The *p*_*i*_’s represent the variance decomposition sensu stricto. In particular, they account for the possibility that, because of covariation, some parameters may offset the contribution of other parameters. The *f*_*i*_’s often more straightforwardly represent the relative role of each parameter because they sum both positive and negative contributions [61]. We report both analyses hereafter for the sake of comparability. The outcomes of both were qualitatively similar in our case.

$$C_{ij}=\rho_{ij}s_{i}\left(\bar{\theta_{i}}\right)s_{j}\left(\bar{\theta_{j}}\right)\text{var}\left(\theta_{i}\right)\text{var}\left(\theta_{j}\right)$$

$$p_{i}=\frac{\sum_{j}C_{ij}}{\sum_{k}\sum_{j}C_{kj}}$$ (3)

$$f_{i}=\frac{\sum_{j}\left|C_{ij}\right|}{\sum_{k}\sum_{j}\left|C_{kj}\right|}$$

### Body mass as a measure of the species-specific rank along the slow–fast continuum of life histories

In several instances, we used body mass to rank species along the slow–fast continuum of life histories instead of a more reliable metric like the generation time [62,63]. This is because our dataset did not include known outliers of the mass–pace of life relationship (i.e., no bats and no mole rats) and because we did not have access to independent generation-time data for all the species in our sample, meaning that using generation time would have introduced statistical correlations between predictors and dependent variables. However, the results did not change qualitatively when using generation time or life span instead of body mass to rank species on the slow–fast continuum.

To assess how the decomposition of life span changed along the slow–fast continuum of life histories [64], we divided our species set in three, according to log-transformed body mass. Thus, 32 species between 132 g and 35.5 kg documented the fast end of the continuum, 32 species between 92 kg and 1.5 ton documented the slow end of the continuum, and 32 species in between documented the medium position. We further assessed whether the between-species variance in the five parameters describing the mortality curve changed from small to large species using a heteroscedasticity test. Namely, we used the studentized Breusch-Pagan heteroscedasticity test [65].

Lastly, we looked for allometric variation in each of the five parameters of Eq 1 using log-log regressions. We used phylogenetic generalized least squares models (PGLSs) [66] for this purpose. PGLSs correct for phylogenetic inertia and thereby remove the phylogenetic signal from dependent variables while estimating the effect of the predictors. Phylogenetic inertia was quantified using the Freckleton’s λ index, which varies from 0 (no phylogenetic signal) to 1 (the variation in the dependent traits is fully accounted by phylogenetic inertia).

### Conformity test

The five parameters of Eq 1 do not vary freely. First, they all need to be positive. Second, they are not independent. For example, if we increase the onset of actuarial senescence while keeping life span constant, then the rate of actuarial senescence needs to increase as well [67]. These mechanistic constraints may have an influence on the variance decomposition. We devised a conformity test to compare our results with the results expected by chance under the effect of these mechanistic constraints. We generated values of the five parameters from independent uniform distributions. We used the observed range of parameter values in our study species to parameterize the uniform distributions, except for the duration of the prime-age period, for which we enforced that the duration could not be longer than the life span of the focal species. We kept generating values until we obtained, for each species in the dataset, 10 simulated mortality curves that had a life span within 1 year of the focal species’ life span. This enforced that the distribution of life span in the resulting simulated datasets was the same as observed in the real dataset. We thereby obtained 500 simulated datasets of 960 species with a life span distribution similar to the observed life span distribution across the 96 species of our dataset. We analyzed each of the simulated datasets in the same way as the real data. This yielded a null-expected distribution for all the quantities that we manipulated, including the decomposition of the variance in life span. If the observed proportion of the variance in life span explained by *θ*_*i*_ matched the expected proportion, with a statistical threshold of 0.05, we concluded that *θ*_*i*_ did not contribute more or less than expected by chance. In the alternative case, *θ*_*i*_ did influence life span more or less than expected by chance.

## Supporting information

S1 Data Raw data from Fig 1. (XLSX)

S2 Data Raw data from Fig 2. (XLSX)

S3 Data Raw data from Fig 3. (XLSX)

S4 Data Raw data from Fig 4. (XLSX)

S5 Data Estimates and standard errors of the five parameters of the age-specific mortality curves for the 96 species, with information on sample size. (XLSX)

S6 Data Details of the model-selection procedure for the 96 species. (XLSX)
